# Supplementary material for: Polyisobutylene—New Opportunities for Medical Applications
Source: Molecules. 2021 Aug 27;26(17):5207. doi: 10.3390/molecules26175207 (PMC8434312; doi:10.3390/molecules26175207)
Supplement: Supplementary file 1 [file molecules-26-05207-s001.zip › molecules-1331829-supplementary.pdf]

Polyisobutylene – New Opportunities for Medical Applications

Dora Barczikai [1], Judit Domokos [2], Dora Szabo [2], Kristof Molnar [3], David Juriga [1], Eniko Krisch [3], Krisztina S. Nagy [1], Laszlo Kohidai [4], Carin A. Helfer [3], Angela Jedlovszky-Hajdu [1,*], and Judit E. Puskas [3,*]

1 Laboratory of Nanochemistry, Department of Biophysics and Radiation Biology, Semmelweis University, Nagyvárad tér 4, Budapest, Hungary 1089; barczikai.dora@gmail.com (D.B.); Juriga.David@med.semmelweis-univ.hu (D.J.); s.nagykriszti@gmail.com (K.S.N.); hajdu.angela@med.semmelweis-univ.hu (A.J-H.)
2 Institute of Medical Microbiology, Semmelweis University, Budapest, Nagyvárad tér 4, Hungary djudit90@gmail.com (J.D.); szabo.dora@med.semmelweis-univ.hu (D.Sz.)
3 Department of Food, Agricultural and Biological Engineering, College of Food, Agricultural, and Environmental Sciences, The Ohio State University, 222 FABE, 1680 Madison Avenue, Wooster, OH 44691; molnar.182@osu.edu(K.M.); molnarnekrisch.1@osu.edu (E.K.); helfer.12@osu.edu (C.A.H.)
4 Semmelweis University, Department of Genetics, Cell- and Immunobiology, Budapest, Nagyvárad tér 4, Hungary 1089 kohlasz2@gmail.com (L.K.)
* Correspondence: puskas.19@osu.edu (J.E.P.); hajdu.angela@med.semmelweis-univ.hu (A.J-H.)

Supplementary information

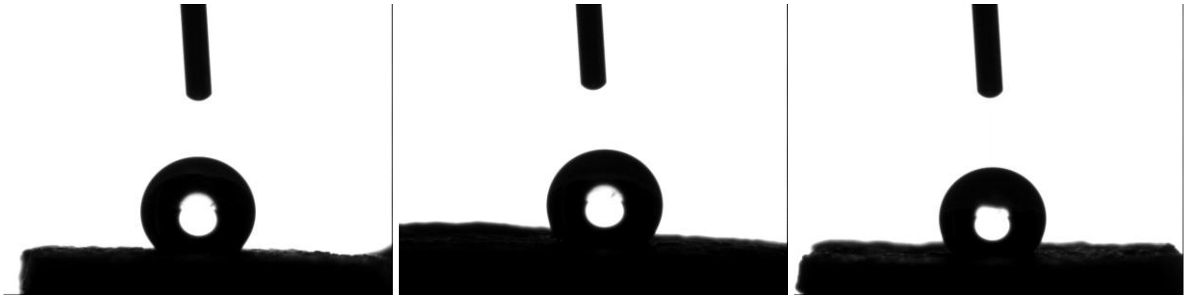

Figure S1. Water contact angle measurement on the 203.75 g/m² mat.

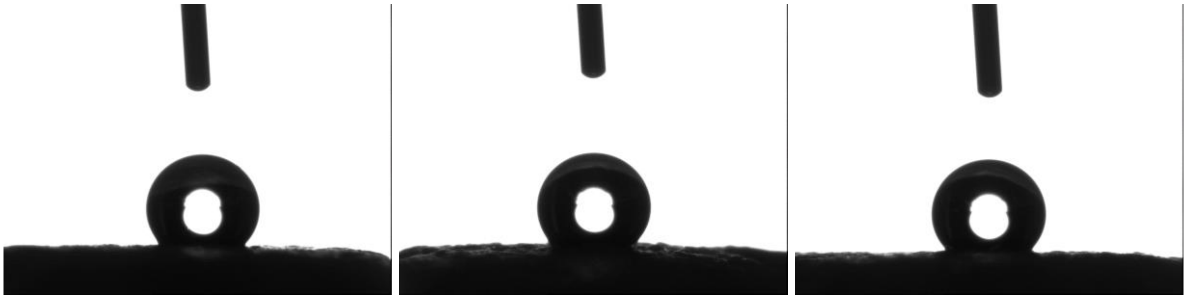

Figure S2. Water contact angle measurement on the 295.5 g/m² mat.
